# Olfactory Sex Recognition Investigated in Antarctic Prions

**DOI:** 10.1371/journal.pone.0004148

**Published:** 2009-01-07

**Authors:** Francesco Bonadonna, Samuel P. Caro, M. de L. Brooke

**Affiliations:** 1 Behavioural Ecology Group, CNRS–CEFE, Montpellier, France; 2 Department of Biology, University of North Carolina, Chapel Hill, North Carolina, United States of America; 3 Department of Zoology, University of Cambridge, Cambridge, United Kingdom; Stockholm University, Sweden

## Abstract

Chemical signals can yield information about an animal such as its identity, social status or sex. Such signals have rarely been considered in birds, but recent results have shown that chemical signals are actually used by different bird species to find food and to recognize their home and nest. This is particularly true in petrels whose olfactory anatomy is among the most developed in birds. Recently, we have demonstrated that Antarctic prions, *Pachyptila desolata*, are also able to recognize and follow the odour of their partner in a Y-maze.

However, the experimental protocol left unclear whether this choice reflected an olfactory recognition of a particular individual (i.e. partner) or a more general sex recognition mechanism. To test this second hypothesis, male and female birds' odours were presented simultaneously to 54 Antarctic prions in a Y-maze. Results showed random behaviour by the tested bird, independent of its sex or reproductive status. Present results do not support the possibility that Antarctic prions can distinguish the sex of a conspecific through its odour but indirectly support the hypothesis that they can distinguish individual odours.

## Introduction

Chemical signals in birds have often been considered a curiosity, supported by limited or anecdotal evidence. However, when tested on particular tasks, almost all bird species have shown abilities for discriminating between olfactory signals [1]–[3]. Among the hardest tasks that birds might achieve by olfactory cues is that of individual recognition. In fact, in vertebrates an olfactory signature often results from a complex bouquet of semiochemicals [i.e. 4] that requires a well-developed olfactory apparatus to be interpreted.

Among birds, petrels (Procellariiforms) have one of the most developed olfactory systems [5], [6]. Olfactory cues appear to be indispensable in several petrel species for locating food [7] and reaching their burrow at night [8]–[14], when visual cues are strongly reduced. In addition, we have recently demonstrated that a petrel species, the Antarctic prion *Pachyptila desolata*, is capable of olfactory partner recognition, the first report of this ability in a bird species [15]. However birds used as odour donors in this experiment were not sexed, leaving open the question whether birds choosing their mate's odour did so because of an individual (i.e. partner) recognition or because of a more general sex recognition/preference. Sex discrimination is well documented in several taxa [16], including mammals [17], [18], and could be expected from bird species that are extremely sensitive to odours [19], [20].

Here we test whether burrowing petrels orient preferentially to odours from the opposite sex when presented with both gender odours in a Y-maze. As social interactions take place mainly at night, often in the darkness of a burrow and silently [21], partly because singing exposes them to predation [22], a complex olfactory communication system, indicating, through their personal odour, information such as their sex, status, quality etc, might be ecologically relevant.

## Methods

The study was conducted on a small sub-Antarctic island (Ile Verte, 49°51′S, 70°05′E) in the Kerguelen Archipelago between January and February 2005, following a similar protocol and employing the same material as used in the experiments of Bonadonna and Nevitt (2004).

Briefly, we presented incubating birds with odour choices in a Y-maze. To trap individual odours, six incubating Antarctic prions (odour donors) were collected from their burrows and held individually in cotton bags (21 by 20 cm; 10 g) for one hour. Bags were then stored separately in ziplock ® plastic storage bags and kept in the dark in a cardboard box for the duration of the experiments (up to 20 days). Bags were stored at ambient temperatures (5–10°C) before being used in experiments. Odour donors were three males and three females whose sex had been genetically determined previously in the Montpellier laboratory following Fridolfsson and Ellegren [23].

Attraction to the scent of bags was tested using a Y-maze with three symmetrical arms (arm length: 60 cm; width: 12 cm; height: 11 cm; angle between each arm ∼120 degrees), made from standard opaque PVC wire housing, and described in detail in Bonadonna and Nevitt (2004). The maze was carefully washed after each trial with methanol (70%) to remove any odour residue. We presented each subject bird with one of nine possible odour pairs, obtained by pairing in turn each female with one of the three available males. The nine pair combinations were used with approximately equal frequency. Odour stimuli were alternated between arms for each trial to eliminate any possible bias between either the choice arms themselves or their spatial positions.

Since the reproductive status of subject birds may influence motivation, we tested both breeding and non-breeding Antarctic prions. Moreover, to guarantee truly blind experiments, the majority of subject birds were of unknown sex at the time of the Y-maze experiments. Overall, subject birds were 14 non-breeders of unknown sex, and 40 incubating birds. The latter group included nine males and 11 females, already genetically sexed as above, and 20 prions of unknown sex. The 34 subject birds of unknown sex across the breeding and non-breeding groups were genetically sexed after the field work [23], and proved to be eight males and six females among non-breeders and ten individuals of each sex among breeders. The breeder group was therefore made of 19 males and 21 females.

In the field, birds were removed from burrows, transported to the maze in a cotton bag (different to the scented bags), placed in the temporary holding compartment of the maze's starting point arm, and allowed to settle for 5 min. At the end of this period a trap door was lifted and the bird was allowed to make a choice in the maze. The choice was easily assessed by the noise of the bird walking in the maze. No-choice birds (removed after 15 min) either never settled down or sat calmly in the holding compartment facing away from the maze arms. We tested whether choice was random using binomial tests.

## Results

The sexes among the categories (breeders and non-breeders) of tested birds and their choices are given in Table 1. We ran binomial tests examining both sex (male or female) and reproductive status (breeder of known sex, or breeder of unknown sex, or non-breeder) categories separately and pooled. None of the tests indicated significant deviation from random choice (Table 1), thus, there was no evidence that the tested birds consistently preferred odour donor of one sex. The proportion of birds making a choice, 46/54 (85.2%: Table 1), was not significantly different to the proportion making a choice, 60/63 (95.2%), in Bonadonna & Nevitt's earlier (2004) series of experiments.

**Table 1:** Choice of each subject bird tested in the Y-maze.

Status	sex of subject bird (n)	choice F	choice M	no choice	*p* value*	♀+♂-*p* value**
(a) breeder, sex known at time of experiment	F (11)	3	6	2	0.25	0.31
	M (9)	4	4	1	0.64	
(b) breeder, sexed subsequent to experiment	F (10)	5	5	0	0.62	0.24
	M (10)	2	6	2	0.14	
(c) non-breeder, sexed subsequent to experiment	F (6)	4	2	0	0.35	0.27
	M (8)	2	3	3	0.5	
(a) & (b)	F (21)	8	11	2	0.32	0.5
	M (19)	6	10	3	0.23	
(a) & (b) & (c)	F (27)	12	13	2	0.5	0.33
	M (27)	8	13	6	0.19	

* *P* values of binomial tests performed for different categories of birds are calculated ignoring birds not making a choice.

** *P* values are calculated on the basis of number preferring own sex versus number preferring opposite sex.

## Discussion

Our present experiments on Antarctic prions did not reveal any preference for odours of birds of either the opposite or the same sex, regardless of the sex or reproductive status of the tested birds. While incubating birds may not have been motivated to distinguish between sexes, either in the present study or in that of Bonadonna and Nevitt [15] the non-breeders tested were presumably birds seeking a mate [21]. However they too showed no sexual preference. Hence, indirectly our present results support the hypothesis that prions can distinguish individual odours, against the concern that experiments of Bonadonna and Nevitt [15] were imperfectly controlled for sex.

To resume the concerns about Bonadonna and Nevitt [15] experiments, we summarize the principal results: (i) in a first experiment 17 out of 20 birds tested in a Y-maze preferred their mate's odour and 3 the odour of a conspecific prion; (ii) in a second experiment out 3 of 20 birds tested preferred their own odour and 17 preferred the odour of a conspecific prion. The authors concluded that Antarctic prions were able to recognize both their own and mate's odours, and that they prefer their mate's odour, but avoid their own odour.

We can assume the birds tested were roughly 50∶50 males and females, and in the first experiment the partners were necessarily of the opposite sex to the subject bird. The observed preference could arise if subject birds preferred the smell of birds of the opposite sex (regardless of whether it was partner) to the smell of a bird of the same sex as the subject bird. More precisely, of the 20 birds tested with their partner's smell, we could envisage 10 might have been faced with a choice between partner and a conspecific of the partner's sex and then showed no preference (5 to partner and 5 to conspecific). The other 10, faced with a choice of partner versus a conspecific of their own sex, would choose the partner (10 to partner). Under this scenario, the outcome would be 15 birds choosing the partner and 5 the conspecific, similar to the observed results of 17∶3. We can apply a similar argument to the second experiment (the own odour is necessarily of the same sex as the test bird). Under a scenario where birds preferred the smell of birds of the opposite sex the outcome would have been 15∶5, again similar to the observed results. Nevertheless, our present results do not show a preference of tested birds for the odour of the opposite sex.

Many petrel species are sexually monomorphic, and meet at breeding colonies in the dark. Thus a sex recognition system that relies on senses other than sight is potentially useful. Acoustic-based communication systems are frequently employed by birds, and may broadcast sex [e.g. 24]. However, calling is a costly activity in some petrel species since avian predators, such as skuas *Catharacta skua lönnbergi*, use calls to localize their petrel prey [22]. Yet acoustic communication is essential during pair formation in many burrowing petrels [21], [25]. Male calling in the burrow attracts females that reply from the air. In many petrel species, including Antarctic prions, there exists a sexual vocal dimorphism [21], [26]. Calls then carry gender information and potentially also information about “quality” [i.e. body condition, 27]. Where the opposite sex can be recognized acoustically, olfactory information may be redundant. However, recent preliminary results on the chemical nature of the personal odours of Antarctic prions show that a sexual dimorphism in the chemical profile might exist [28], The absence of significant behavioural results presented here does not exclude the possibility that smell may be a potential signal of sex in birds [29], [30]. For example it may contribute to a synergism of acoustic and olfactory cues in a multimodal strategy transferring the information “sex”.

The present results validate the conclusions of Bonadonna and Nevitt [15] showing that, in the Y-maze setup used, Antarctic prions were not driven by a sexual preference but rather by a preference for the odour of a partner or a conspecific. However the question of whether Antarctic prions recognise the sex of an individual through its odour too is still unanswered. Smell, potentially a signal of sex, may play a more important role in birds' social lives than generally recognized, in particular in mate choice. Nevertheless, we actually do not know if olfaction play a role in pair formation, a fascinating possibility that still belongs in the realm of speculation [but see 15], [but see 31], [32].
